# Methodological and reporting quality evaluation of meta-analyses on the Chinese herbal preparation Zheng Qing Feng Tong Ning for the treatment of rheumatoid arthritis

**DOI:** 10.1186/s12906-020-02978-5

**Published:** 2020-06-26

**Authors:** Mingge Liang, Lan Yan, Zhigang Mei, Yanan Luo, Xiaoqiang Hou, Zhitao Feng

**Affiliations:** 1 Third-Grade Pharmacological Laboratory on Chinese Medicine Approved by State Administration of Traditional Chinese Medicine, Medical College of China Three Gorges University, Yichang, 443002 Hubei China; 2 The Key Laboratory of Hunan Province for Integrated Traditional Chinese and Western Medicine on Prevention and Treatment of Cardio-Cerebral Diseases, Hunan University of Chinese Medicine, Changsha, 410208 Hunan China; 3 Institute of Rheumatology, the First College of Clinical Medical Sciences, China Three Gorges University, Yichang, 443003 Hubei China

**Keywords:** Sinomenine, Zheng Qing Feng Tong Ning, Rheumatoid arthritis, Meta-analysis, Methodology, Reporting quality

## Abstract

**Background:**

Zheng Qing Feng Tong Ning (ZQFTN) is a sinomenine (SIN) preparation that has been used in clinical practice. Our study aimed to assess the methodological and reporting quality of meta-analyses on the Chinese herbal formula ZQFTN for the treatment of rheumatoid arthritis (RA).

**Methods:**

Systematic searches were carried out with the 5 following electronic databases from inception to July 2019: China National Knowledge Infrastructure (CNKI), Wanfang, VIP database for Chinese technical periodicals (VIP), Cochrane Library and PubMed. The quality of the methodology and reporting was measured with the assessment of multiple systematic reviews 2 (AMSTAR 2) scale, the Preferred Reporting Items for Systematic Reviews and Meta-Analyses (PRISMA) statement and the Grading of Recommendations, Assessment, Development and Evaluation (GRADE).

**Results:**

Eight studies were identified. Among the 16 items of the AMSTAR 2 scale, four items were optimally reported (“Y” =100% of the items), and another four items were poorly reported (“Y” =0% of the items). Only 2 studies received a good overall score (“Y” ≥50% of the items). Regarding the PRISMA statement, the scores of 5 studies were lower than the average score (17.69), indicating that the quality of the reports was very low. In terms of the GRADE, none of the 61 results were of high quality (0.0%). Fifteen results were of medium quality (25%), 34 were of low quality (55%), and 12 were of very low quality (20%). Among the five downgrading factors, deviation risk (*n* = 61, 100%) was the most common downgrading factor, followed by inconsistency (*n* = 30, 50%), publication bias (*n* = 17, 28%), inaccuracy (*n* = 11, 18%) and indirectness (*n* = 0, 0%).

**Conclusions:**

The methodological and reporting quality of the meta-analyses and systematic reviews in the included studies are less than optimal, and researchers should undergo additional training and follow the AMSTAR 2 scale, PRISMA statement and GRADE to design high-quality studies in the future.

## Background

Rheumatoid arthritis (RA) is a systemic inflammatory autoimmune disease that may trouble patients as a result of morning stiffness, painful joints, chronic inflammation, synovitis, irrecoverable joint damage, and the presence of autoantibodies [1, 2]. The prevalence of RA in adults worldwide is 0.04–1.6%, with significant national differences [3]. In China, RA has an estimated prevalence of 0.42% and affected more than 5 million patients in 2018 [4]. The pathogenesis of RA is complex, and the course of RA is lingering; RA is characterized by symmetrical, chronic, and progressive polyarthritis, which, as the disease progresses, leads to the destruction of articular cartilage, bone, and capsule, resulting in irreversible joint deformity and incapacitation [5, 6]. At present, the common medications for RA include glucocorticoids (GCs), nonsteroidal anti-inflammatory drugs (NSAIDs), and disease-modifying antirheumatic drugs (DMARDs) [7–9]. Some studies have reported that sinomenine (SIN), *Tripterygium wilfordii* Hook, Simiao pill, Wang-bi tablet, total glucosides of paeony (TGP) [10–15] and other traditional Chinese medicines and their related prescriptions possess beneficial effects and show good clinical efficacy in the treatment of RA, supporting why traditional Chinese medicines and prescriptions have received increasing attention [16–18].

Zheng Qing Feng Tong Ning (ZQFTN) is one of the SIN preparations, and it is an alkaloid monomer extracted from the traditional Chinese herb *Sinomenium acutum* and has been used in clinical practice [19]. Some studies have shown that SIN may have a good effect on the treatment of RA (e.g., less pain and an improvement in physical function or morning stiffness) [20, 21]. Mechanistic studies have indicated that SIN can alleviate collagen-induced arthritis (CIA) via the inhibition of angiogenesis [22], induce the generation of intestinal Treg cells, relieve arthritis by activating the aryl hydrocarbon receptor [﻿23﻿] and suppress RA progression by modulating the secretion of various inflammatory cytokines and the monocyte/macrophage subpopulation [﻿24﻿]. Currently, ZQFTN series products are one of the Chinese medicine varieties used for the domestic treatment of RA, and ZQFTN is a modern Chinese medicine preparation [﻿25﻿]. Studies have shown that SIN has anti-inflammatory, analgesic and immunosuppressive effects [﻿26﻿], which indicates that it may play a crucial role in the treatment of RA. A multitude of clinical trials on the efficacy and safety of ZQFTN in the treatment of RA have been performed in mainland China and other countries. The relevant methods and quality analyses of the reports may promote the evidence-based clinical treatment of RA. Systematic limitations or deficiencies in the design, conduct, or report of articles may bias the results.

The assessment of multiple systematic reviews (AMSTAR) is a tool used for the rigorous evaluation of systematic reviews of randomized controlled clinical trials that explicitly focuses on assessing risk of bias (RoB) and internal effectiveness in the methodological quality of intervention-related systemic resuscitation [﻿27﻿]; the Preferred Reporting Items for Systematic Reviews and Meta-Analyses (PRISMA) is a reporting guideline that has made some advances in concepts and methods in randomized trials that conduct and report systematic reviews [﻿28﻿]; and the Grading of Recommendations, Assessment, Development and Evaluation (GRADE) approach is more reliable than intuitive judgments when assessing the quality of evidence on outcomes of health care interventions [﻿29﻿]. However, until now, there has been no systematic review that explored the characteristics associated with the methodological quality of controlled trials (random or nonrandom) that evaluated the effectiveness and safety of ZQFTN in the treatment of RA. Therefore, we searched all systematic reviews and meta-analyses of SIN and its preparations in RA until 2019 and applied three tools, AMSTAR 2, PRISMA and GRADE, to evaluate the quality of these studies. Ultimately, the aim of our study was to provide better evidence-based medical support for the clinical application of SIN in RA.

## Methods

### Search strategy

Systematic searches were carried out in the China National Knowledge Infrastructure (CNKI), Wanfang, VIP database for Chinese technical periodicals (VIP), Cochrane Library and PubMed databases through the end of July 2019. The Medical Subject Headings (MeSH) items included “sinomenine”, “sinomenine preparation”, “Zhengqing Fengtongning”, “RA”, “rheumatoid arthritis”, “meta-analysis” and “systematic review”. The keywords contained “Qing teng jian”, “Qing teng jian zhi ji”, “Zheng qing feng tong ning”, “Lei feng shi guan jie yan”, “Lei feng shi xing guan jie yan”, “meta fen xi”, “Xi tong ping jia” and “Hui cui fen xi” (in Chinese). The detailed search strategy is shown in supplementary Tables 1 and 2.

### Selection of reviews

The inclusion criteria were as follows: (1) article types were systematic reviews and meta-analyses; (2) the drug intervention was SIN, SIN preparations, ZQFTN, or ZQFTN sustained-release tablets; (3) studies that utilized the RA classification standards established by the American College of Rheumatology (ACR) in 1987; (4) articles published in English or Chinese; and (5) studies published in journals.

The exclusion criteria were as follows: (1) studies were neither systematic reviews nor meta-analyses; (2) the drug intervention was neither SIN nor ZQFTN; (3) the sample included patients with other diseases; (4) systematic reviews/meta-analyses theory or literature quality; (5) a republished article or an article not published in full; and (6) academic dissertations or conference papers.

### Document selection and data extraction

Excel 2010 software was used to establish AMSTAR 2, PRISMA and GRADE evaluation scales. Two reviewers completed the literature retrieval independently, screening according to the inclusion and exclusion criteria, and extracted the data according to the preestablished forms. The extracted data were as follows: basic information (studies, publication year, language, publication form, number of documents, and number of cases), intervention measures (experimental group vs. control group), outcome, and conclusion. Any disagreement was resolved by discussion with a third party (Zhitao Feng).

### Quality assessment

The AMSTAR 2 scale and PRISMA statement were used for the methodological and reporting evaluation, respectively, and the GRADE was used for the evidence quality evaluation [27–29]. The evaluation scales were preassigned by Excel 2010. Two reviewers completed the evaluation of the quality of the literature independently. The literature was also evaluated by the AMSTAR 2 scale, PRISMA statement, and GRADE. The rating criteria were as follows.

The AMSTAR 2 scale comprises 16 items. If the item is adequately answered and correct, it is judged as “Yes”. If the item is answered correctly but the evidence is insufficient, it is judged as “Partial Yes”. If there is no information in the article, it is judged as “No”. Answers of “Yes” are scored as 1 point, and answers of “No” and “Partial Yes” receive no score; the total score is 11 points.

The PRISMA statement contains 27 items, and each item is scored as follows: a complete report scores 1 point, a partial report scores 0.5 points, and no report scores 0 points. When the score is 21–27, the report is considered relatively complete; when the score is 15–21, the report is considered to have certain defects; and when the score is below 15, relatively serious information is considered to be missing.

The five downgrading elements of the GRADE were as follows: RoB (unrepresentative sample, allocation concealment, not blinded, incomplete reporting of patient and outcome events, and selective results reporting bias and other limitations), indirectness (indirect comparison of the population, intervention, comparator, and outcome (PICO)), inconsistency (similarity of point estimates, overlap degree of confidence intervals (CIs), heterogeneity test *P* < 0.05, and heterogeneity *I*^*2*^ > 50%), imprecision (small sample size and a wide 95% CI) and publication bias (funnel plots, Egger test, including unpublished research and gray literature). The quality of evidence is divided into four levels by the GRADE: high (we have great confidence that the real effect is close to the estimated result), moderate (we have moderate belief that the actual effect is close to the estimated result), low (we have limited confidence in the effectiveness estimate), and very low (we have little confidence that the actual results are comparable to the estimated results). Initially, each result defaults to “high” quality and is classified into the above 4 levels after a judgment of the 5 downgrading factors. Two reviewers carefully studied each evaluation scale and agreed on the evaluation criteria, and then each reviewer performed an independent literature evaluation. In the case of a disagreement, a third party (Zhitao Feng) discussed the decision to reach an agreement.

## Results

### Results of the search strategy

**Fig. 1:**
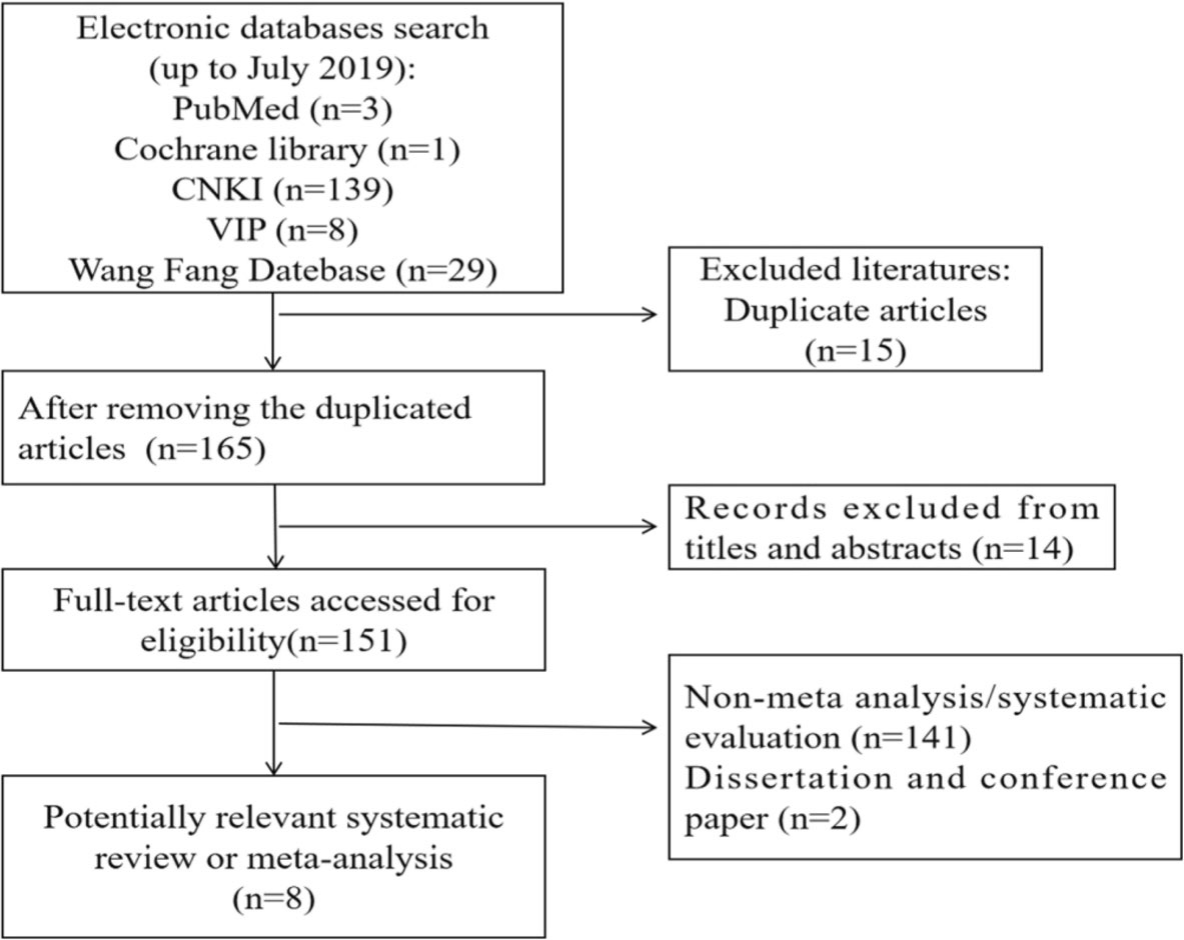
Flow chart of literature search. Abbreviation: CNKI, China National Knowledge Infrastructure; VIP, VIP Database for Chinese Technical Periodicals

The initial search yielded 180 articles, of which 15 were excluded because they were duplicates, and 14 were excluded after reading the titles and abstracts. Of the remaining 151 articles, 143 were excluded because they did not meet the inclusion criteria after the full-text screen. Finally, 8 articles were accepted: 5 published in Chinese and 3 published in English. The screening process is summarized in a flow diagram in Fig. 1, and the basic information of the included studies is shown in Table 1.

**Table 1:** The general information of the included studies

Study	Language	Publication form	Number of documents (case number)	Intervention measures (control vs treatment)	Outcomes	AMSTAR2 (point)	PRIMSA (point)
Xu 2008 [20]	English	Journal	10 (1185)	SIN vs single NSAIDs; SIN vs combined NSAIDs	(1) NIP; (2) NRP; (3) MS; (4) PJ; (5) SJ; (6) GS; (7) ESR; (8) CPR; (9) JTS; (10) AI	6	17
Qi 2010 [﻿30﻿]	Chinese	Journal	3 (280)	ZQFTN vs MTX;	(1) MS; (2) SJ; (3) PJ; (4) ESR; (5) RF;	4	16
Zhang 2012 [﻿31﻿]	Chinese	Journal	10 (1365)	NSAIDs + MTX + ZQFTN vs NSAIDs + MTX; MTX + ZQFTN vs MTX; NSAIDs + MTX + PED + ZQFTN vs NSAIDs + MTX + PED; MTX + SSZ + ZQFTN vs MTX + SSZ; LEF + MTX + ZQFTN vs LEF + MTX;	(1) ACR; (2) PJ; (3) SJ; (4) ESR; (5) CPR; (6) RF;	7	17.5
Li 2012 [﻿32﻿]	Chinese	Journal	8 (735)	ZQFTN + MTX vs MTX	(1) Total Effect; (2) MS; (3) ESR; (4) RF; (5) CRP; (6) Adverse Effects	5	15.5
Wang 2015 [﻿33﻿]	Chinese	Journal	6 (476)	ZQFTN + NSAIDs + MTX vs NSAIDs + MTX; ZQFTN vs OXA; ZQFTN + SSZ vs MTX + SSZ; ZQFTN + MTX vs MTX; ZQFTN + MTX vs MTX + D-Pen; ZQFTN + NSAIDS + MTX vs NSAIDs + MTX;	(1) Total Effect; (2) Adverse Effects; (3) MS; (4) ESR; (5) CPR; (6) RF;	8	19
Chen 2015 [﻿34﻿]	English	Journal	11 (956)	ZQFTN+MTX vs MTX;	(1) RF; (2) CPR; (3) ESR; (4) SJ; (5) MS	6	16
Li 2016 [﻿35﻿]	Chinese	Journal	8 (708)	NSAIDS + ZQFTN + MTX vs NSAIDs + MTX; ZQFTN + SSZ vs MTX + SSZ; ZQFTN + MTX vs MTX; ZQFTN + MTX vs MTX + D-Pen; ZQFTN + LEF + NSAIDS vs LEF + NSAIDs	(1) Total Effect; (2) Adverse Effects; (3) MS; (4) PJ; (5) GS; (6) SJ; (7) ESR; (8) RF; (9) CPR;	7	20.5
Liu 2016 [21]	English	Journal	16 (1500)	ZQFTN vs MTX; ZQFTN + MTX vs MTX; Basic therapy + ZQFTN + MTX vs Basic therapy + MTX	(1) Clinical efficacy; (2) MS; (3) Total clinical effective rate in 4 weeks treatment; (4) SJ; (5) GS; (6) ESR; (7) CPR; (8) Blood platelet; (9) DAS28;	10	20

*Abbreviations*: *SIN* Sinomenine preparations, *NSAIDs* Non-steroidal anti-inflammatory drugs, *NIP* Number of improved patients, *NRP* Number of rheumatoid-factor-disappeared patients, *MS* Morning stiffness, *PJ* Painful joint, *SJ* Swollen joint, *GS* Grip strength, *ESR* Erythrocyte sedimentation rate, *CRP* C-reactive protein, *JTS* Joint tenderness score, *AI* Articular index, *ZQFTN* Zhengqing Fengtongning release tablets, *MTX* Methotrexate, *LEF* Leflunomide, *D-pen* D-penicillamine, *SSZ* Sulfasalazine, *PED* Prednisone, *ACR* American college of rheumatology, *RF* Rheumatoid factor, *DAS28* Disease activity score-28

### Literature analysis

#### Amstar 2

The average AMSTAR 2 score was 6.625 (full score 16); the highest score was 10, and the lowest score was 4 (Table 1). Only two included studies achieved a good overall AMSTAR 2 score (“Y” ≥ 50% of the items) [21, 33], and the optimal items (8/8) were item 1, item 5, item 6 and item 8. All of the studies adequately used the PICO components. Five (5/8) [20, 21, 32, 33, 35] reviews appropriately explained the sources of funding. Five (5/8) [20, 21, 31, 34, 35] reviews accounted for RoB in the primary studies. Three (3/8) [21, 33, 35] studies assessed the potential impact of RoB in individual studies on the results and investigated the publication bias sufficiently. Only two (2/8) [30, 32] studies evaluated RoB using an acceptable technique and provided a satisfactory explanation for and discussion of any observed heterogeneity. A comprehensive literature search strategy is necessary; however, it appeared only in one (1/8) [21] review. Any missing reports regarding a conflict of interest could mislead researchers, and only one (1/8) [20] study mentioned this topic. None (0/8) of the reviews mentioned item 2, item 3, item 7 or item 11, and a statement regarding the review methods being established primarily, the selection of the studies for inclusion, the appropriate methods for the statistical combination of results, and a list of excluded studies were all lacking (Table 2).

**Table 2:** AMSTAR 2 scores for the methodology of reviewers included in study

Study	Item 1	Item 2	Item 3	Item 4	Item 5	Item 6	Item 7	Item 8	Item 9	Item 10	Item 11	Item 12	Item 13	Item 14	Item 15	Item 16	Of “Y”
Xu 2008 [20]	Y	N	N	P	Y	Y	N	Y	P	Y	N	N	Y	N	N	N	6 (37.50%)
Qi 2010 [﻿30﻿]	Y	N	N	P	Y	Y	N	Y	P	N	N	N	N	N	N	N	4 (25.00%)
Zhang 2012 [﻿31﻿]	Y	N	N	P	Y	Y	N	Y	P	N	N	N	Y	Y	Y	N	7 (43.75%)
Li 2012 [﻿32﻿]	Y	N	N	P	Y	Y	N	Y	P	Y	N	N	N	N	N	N	5 (31.25%)
Wang 2015 [﻿33﻿]	Y	N	N	P	Y	Y	N	Y	P	Y	N	Y	N	Y	Y	N	8 (50.00%)
Chen 2015 [34]	Y	N	N	P	Y	Y	N	Y	Y	N	N	N	Y	N	N	Y	6 (37.50%)
Li 2016 [﻿35﻿]	Y	N	N	P	Y	Y	N	Y	P	Y	N	Y	Y	N	N	N	7 (43.75%)
Liu 2016 [21]	Y	N	N	Y	Y	Y	N	Y	Y	Y	N	Y	Y	N	Y	N	10 (62.50%)
Of “Y”	8 (8/8)	0 (0/8)	0 (0/8)	1 (1/8)	8 (8/8)	8 (8/8)	0 (0/8)	8 (8/8)	2 (2/8)	5 (5/8)	0 (0/8)	3 (3/8)	5 (5/8)	2 (2/8)	3 (3/8)	1 (1/8)	

Item 1: did the research questions and inclusion criteria for the review include the components of PICO? Item 2: did the report of the review contain an explicit statement that the review methods were established prior to the conduct of the review and did the report justify any significant deviations from the protocol? Item 3: did the review authors explain their selection of the study designs for inclusion in the review? Item 4: did the review authors use a comprehensive literature search strategy? Item 5: did the review authors perform study selection in duplicate? Item 6: did the review authors perform data extraction in duplicate? Item 7: did the review authors provide a list of excluded studies and justify the exclusions? Item 8: did the review authors describe the included studies in adequate detail? Item 9: did the review authors use a satisfactory technique for assessing the risk of bias (RoB) in individual studies that were included in the review? Item 10: did the review authors report on the sources of funding for the studies included in the review? Item 11: if meta-analysis was performed, did the review authors use appropriate methods for statistical combination of results? Item 12: if meta-analysis was performed, did the review authors assess the potential impact of RoB in individual studies on the results of the meta-analysis or other evidence synthesis? Item 13: did the review authors account for RoB in primary studies when interpreting/discussing the results of the review? Item 14: did the review authors provide a satisfactory explanation for, and discussion of, any heterogeneity observed in the results of the review? Item 15: if they performed quantitative synthesis did the review authors carry out an adequate investigation of publication bias (small study bias) and discuss its likely impact on the results of the review? Item 16: did the review authors report any potential sources of conflict of interest, including any funding they received for conducting the review?

*Abbreviations: Y* “Yes”, *P* “Partial Yes”, *N* “No”

#### PRISMA

The average PRISMA score was 17.69 (maximum score 27). The maximum score of the eight included articles was 20.5, and the minimum score was only 15.5, as shown in Table 1. None of the articles reported the 27 items completely. (1) Title: All articles reported the title (8/8); (2) Structured summary: Two papers did not meet the criteria of providing structured abstracts; neither of them reported the background of the study nor the registration number of the study [20, 34]. (3) Introduction: All the studies described the theoretical basis in detail and reported the purpose completely, but no complete report on previous reviews was provided. (4) Methods: None of the documents reported registration information or complete report plans. None of the corresponding gray literature was selected. Only one of the studies completely reported a database search strategy [﻿21﻿]. In the course of describing the selected studies, 4 studies reported a PRISMA literature screening flow chart [21, 33–35]. Four papers reported RoB in individual studies but did not describe how bias was used to evaluate the results or its impact on outcomes in further studies [21, 31, 33, 35]. Only 3 studies reported publication bias (i.e., funnel charts were drawn) [21, 33, 35]. All of the studies listed the characteristics of the included studies in detail and tested for homogeneity and heterogeneity. (5) Results: None of the articles fully described the characteristics of the studies or reported the follow-up time, funding resources, etc. Two papers did not fully report the study selection [20, ﻿30﻿] and failed to provide the reasons for excluding the literature at each step. Eight papers described the results of individual studies and results in the synthesis and carried out homogeneity and heterogeneity tests. Only 1 article [21] explained other analyses, such as subgroup analysis and sensitivity analysis. (6) Discussion: Five articles [20, 30, 31, 33, 35] used graphs to demonstrate each major result, and only 1 article [﻿32﻿] did not report the limitations of the systematic review. (7) Funding: Five articles reported funding sources [20, 21, 32, 33, 35], but only 1 mentioned the role of the funders [20] (Table 3).

**Table 3:** Reporting quality analysis of Meta-analyses of SIN treatment of RA

	PRISMA Item	Adequate	Partial	Inadequate.
Title	Title	8	0	0
Abstract	Structured summary	0	6	2
Introduction	Rationale	8	0	0
	Objectives	8	0	0
Methods	Protocol and registration	0	0	8
	Eligibility criteria	8	0	0
	Information sources	8	0	0
	Search	1	7	0
	Study selection	1	7	0
	Data collection process	8	0	0
	Data items	0	0	8
	Risk of bias in individual studies	2	2	4
	Summary measures	8	0	0
	Synthesis of results	8	0	0
	Risk of bias across studies	2	2	4
	Additional analyses	4	2	2
Results	Study selection	6	0	2
	Study characteristics	0	8	0
	Risk of bias within studies	0	4	4
	Results of individual studies	8	0	0
	Synthesis of results	8	0	0
	Risk of bias across studies	3	1	4
	Additional analysis	1	0	7
Discussion	Summary of evidence	3	2	3
	Limitations	7	0	1
	Conclusions	8	0	0
Funding	Funding	1	4	3

#### Grade

Sixty-one outcomes measured by the 8 included reviews. Among these outcomes, high quality of evidence was found in none of the reviews (0.0%), moderate evidence was found in 15 reviews (25%), low evidence was found in 34 reviews (55%), and very low evidence was found in 12 reviews (20%). Regarding the five downgrading elements, the most common items were RoB (*n* = 61, 100%), inconsistency (*n* = 30, 50%), publication bias (*n* = 17, 28%), imprecision (*n* = 11, 18%) and indirectness (*n* = 0, 0%) (Table 4).

**Table 4:** GRADE for quality of evidence profile

Study ID	Outcomes (number of studies)	Risk of bias	Inconsistency	Indirectness.	Imprecision	Publication bias	Quality of evidence
Xu 2008 [20]	NIP (10)	Serious^a^	Not serious	Not serious	Not serious	Undetected	Moderate
	NRP (4)	Serious^a^	Not serious	Not serious	Not serious	Undetected	Moderate
	MS (3)	Serious^a^	Not serious	Not serious	Not serious	Undetected	Moderate
	PJ (3)	Serious^a^	Serious^c^	Not serious	Not serious	Undetected	Low
	ESR (4)	Serious ^a^	Not serious	Not serious	Not serious	Undetected	Moderate
	SJ (4)	Serious ^a^	Serious^c^	Not serious	Not serious	Undetected	Low
	GS (3)	Serious ^a^	Serious^c^	Not serious	Not serious	Undetected	Low
	CPR (3)	Serious ^a^	Not serious	Not serious	Serious^d^	Undetected	Low
	ADEs (4)	Serious ^a^	Not serious	Not serious	Serious^d^	Undetected	Low
Qi 2010 [﻿29﻿]	MS (3)	Serious ^a^	Not serious	Not serious	Serious^d^	Undetected	Low
	SJ (3)	Serious ^a^	Serious^c^	Not serious	Serious^d^	Undetected	Very low
	PJ (2)	Serious ^a^	Not serious	Not serious	Serious^d^	Undetected	Low
	ESR (2)	Serious ^a^	Not serious	Not serious	Serious^d^	Undetected	Low
	RF (2)	Serious ^a^	Serious^c^	Not serious	Serious^d^	Undetected	Very low
Zhang 2012 [﻿30﻿]	ACR (2)	Serious ^a^	Serious^c^	Not serious	Not serious	Strongly suspected^b^	Very low
	PJ (8)	Serious ^a^	Serious^c^	Not serious	Not serious	Strongly suspected^b^	Very low
	SJ (8)	Serious ^a^	Serious^c^	Not serious	Not serious	Strongly suspected^b^	Very low
	ESR (9)	Serious ^a^	Serious^c^	Not serious	Not serious	Strongly suspected^b^	Very low
	CPR (7)	Serious ^a^	Not serious	Not serious	Not serious	Strongly suspected^b^	Low
	RF (9)	Serious ^a^	Not serious	Not serious	Not serious	Strongly suspected^b^	Low
Li 2012 [﻿31﻿]	Total Effect (6)	Serious ^a^	Not serious	Not serious	Not serious	Strongly suspected^b^	Low
	MS (3)	Serious ^a^	Not serious	Not serious	Serious^d^	Strongly suspected^b^	Very low
	ESR (5)	Serious ^a^	Not serious	Not serious	Not serious	Strongly suspected^b^	Low
	RF (5)	Serious ^a^	Not serious	Not serious	Not serious	Strongly suspected^b^	Low
	CRP (8)	Serious ^a^	Not serious	Not serious	Not serious	Strongly suspected^b^	Low
Wang 2015 [﻿32﻿]	Total Effect (6)	Serious ^a^	Not serious	Not serious	Not serious	Strongly suspected^b^	Low
	MS (5)	Serious ^a^	Serious^c^	Not serious	Not serious	Strongly suspected^b^	Very low
	ESR (6)	Serious ^a^	Serious^c^	Not serious	Not serious	Strongly suspected^b^	Very low
	RF (6)	Serious ^a^	Serious^c^	Not serious	Not serious	Strongly suspected^b^	Very low
	CPR (6)	Serious ^a^	Not serious	Not serious	Serious^d^	Strongly suspected^b^	Very low
	ADEs (5)	Serious ^a^	Not serious	Not serious	Not serious	Strongly suspected^b^	Low
Chen 2015 [34]	Total Effect of ZQFTN (11)	Serious ^a^	Serious^c^	Not serious	Not serious	Undetected	Low
	RF (8)	Serious ^a^	Serious^c^	Not serious	Not serious	Undetected	Low
	ESR (10)	Serious ^a^	Serious^c^	Not serious	Not serious	Undetected	Low
	CRP (8)	Serious ^a^	Serious^c^	Not serious	Not serious	Undetected	Low
	D MS (6)	Serious ^a^	Serious^c^	Not serious	Not serious	Undetected	Low
	SJC (6)	Serious ^a^	Not serious	Not serious	Not serious	Undetected	Moderate
	TGC (7)	Serious ^a^	Not serious	Not serious	Not serious	Undetected	Moderate
	ADEs (10)	Serious ^a^	Not serious	Not serious	Not serious	Undetected	Moderate
Li 2016 [﻿33﻿]	Total Effect of ZQFTN (8)	Serious ^a^	Not serious	Not serious	Not serious	Undetected	Moderate
	MS (8)	Serious ^a^	Serious^c^	Not serious	Not serious	Undetected	Low
	PJ (8)	Serious ^a^	Serious^c^	Not serious	Not serious	Undetected	Low
	GS (8)	Serious ^a^	Serious^c^	Not serious	Not serious	Undetected	Low
	SJ (8)	Serious ^a^	Serious^c^	Not serious	Not serious	Undetected	Low
	ESR (8)	Serious ^a^	Serious^c^	Not serious	Not serious	Undetected	Low
	RF (8)	Serious ^a^	Serious^c^	Not serious	Not serious	Undetected	Low
	CPR (8)	Serious ^a^	Serious^c^	Not serious	Not serious	Undetected	Low
	AD (8)	Serious ^a^	Not serious	Not serious	Not serious	Undetected	Moderate
Liu 2016 [21]	Clinical efficacy (15)	Serious ^a^	Not serious	Not serious	Not serious	Undetected	Moderate
	Publication bias (15)	Serious ^a^	Not serious	Not serious	Not serious	Undetected	Moderate
	Subgroup analysis (15)	Serious ^a^	Not serious	Not serious	Not serious	Undetected	Moderate
	Sensitivity analysis (15)	Serious ^a^	Not serious	Not serious	Not serious	Undetected	Moderate
	Total clinical effective rate in 4 weeks treatment (2)	Serious ^a^	Serious^c^	Not serious	Serious^d^	Undetected	Very low
	MS (12)	Serious ^a^	Serious^c^	Not serious	Not serious	Undetected	Low
	SJ (9)	Serious ^a^	Serious^c^	Not serious	Not serious	Undetected	Low
	GS (6)	Serious ^a^	Serious^c^	Not serious	Not serious	Undetected	Low
	ESR (12)	Serious ^a^	Serious^c^	Not serious	Not serious	Undetected	Low
	CRP (11)	Serious ^a^	Serious^c^	Not serious	Not serious	Undetected	Low
	PLT (2)	Serious ^a^	Not serious	Not serious	Serious^d^	Undetected	Low
	DAS28(4)	Serious ^a^	Not serious	Not serious	Not serious	Undetected	Moderate
	ADEs (12)	Serious ^a^	Not serious	Not serious	Not serious	Undetected	Moderate

*Abbreviations***:***SIN* Sinomenine preparations, *NSAIDs* Non-steroidal anti-inflammatory drugs, *NIP* Number of improved patients, *NRP* Number of rheumatoid-factor-disappeared patients, *MS* Morning stiffness, *PJ* Painful joint, *SJ* Swollen joint, *SJC* Swollen Joint Count, *GS* Grip strength, *ESR* Erythrocyte sedimentation rate, *CRP* C-reactive protein, *AD* Adverse effects, *JTS* Joint tenderness score, *AI* Articular index, *ZQFTN* Zhengqing Fengtongning release tablets, *MTX* Methotrexate, *DMS* Duration of morning stiffness, *TGC* Tender Joint Count, *ADEs* Adverse Effects, *PLT* Blood platelet, *DAS28* Disease activity score for rheumatoid arthritis in 28 Joints

^a^(Unclear random sequence generation, allocation concealment blinding not done in all studies)

^b^(Incomplete retrieval for unpublished studies and gray literature, evidence for publication bias was underpowered)

^c^(The overlap degree of different research confidence intervals is poor, and *I*^*2*^ > 50%)

^d^(Inadequate sample size and the wide 95% (CI))

## Discussion

It is important to assess the methodological quality and quality of evidence of systematic reviews/meta-analyses in the field of evidence-based medicine before any conclusions can be reached for clinical decision making [﻿36﻿, 37]. Reviews with qualified methodologies and high quality of evidence can provide comprehensive and reliable evidence for decision-makers [﻿38﻿]. This study is the first to evaluate the methodological and reporting quality of meta-analyses or systematic reviews on SIN and its preparation, ZQFTN, in the treatment of RA, intending to improve the quality of systematic reviews and better guide clinical decisions. In addition to AMSTAR 2, PRISMA was also used, and the GRADE was used to assess the quality of evidence for the outcome of RA interventions with SIN or ZQFTN. This study will help improve the quality of systematic reviews/meta-analyses and provide an intuitive judgment on the clinical efficacy of SIN and ZQFTN on RA. Concerning the quality of the eight articles we included, unfortunately, the results revealed some limitations in the quality of methodology and reporting, suggesting the need for an improvement in quality in the future.

In summary, only a mean of 42% of AMSTAR 2 items were fulfilled across all articles. The major defects found are described as follows: first, there was no mention of whether the systematic evaluation method was predetermined, there was no complete explanation of the type of study design, and the list of excluded studies was not provided, which may be related to layout restrictions; second, the appropriate statistical methods were not used for the combined analysis of the results; and more than half of the reviews mentioned financial support for inclusion, but only a small proportion explained its function and clarified conflicts of interest in detail. The impact of the RoB of each included study on outcomes, the heterogeneity of the results, and publication bias were limited. All of these are important for readers to accurately assess the methods and results.

However, we found that the reporting was of poor quality, and the Chinese literature scores were generally lower than those of the English literature; some of these low scores were the result of underreporting or a lack of information. No registration number was provided, and only one of the studies provided a complete report of the database search strategy used [21]. The individual research bias of four studies was absent [20, 30, 32, 34], the publication bias of four studies was absent [20, 30–32], and the selection bias of three studies was absent [21, 33, 35], all of which should be described and analyzed. There was a lack of detailed information on financial support [30, 31, 34] and the role of the funder in the study [21, 32, 33, 35]. A failure to report such information may increase bias and reduce the authenticity and reliability of the research. Therefore, the results of this study may have been underestimated due to a lack of important information. We strongly recommend that editors and authors recognize and promote the use of reporting guidelines in their publications.

In addition, we found that 75% of the outcome indicators had a low or very low quality of evidence in the GRADE table, indicating that the true effect might be substantially different from the estimated effect in these reviews. Of the five downgrading factors, RoB was the most common factor that reduced the level of evidence. This indicates that we should pay close attention to assignment hiding, blinding methods and selective reporting to reduce the impact of limitations on outcome indicators. Because the overlap degree of different research CIs was poor and *I*^2^ > 50%, the inconsistency of the result indicators was reduced. This inaccuracy is mostly due to insufficient sample sizes and a wide 95% CI, which indicates that the sample size and sample advisability should receive more attention. Regarding publication bias, most of the included literature did not carry out specific tests or analyses, mostly because of the lack of gray literature and statistical tests showing insufficient momentum, resulting in reduced quality. Therefore, in future research on ZQFTN or SIN for the treatment of RA, researchers need to pay close attention to the quality of evidence of outcome indicators and provide readers with the highest possible quality of evidence indicators.

Research has revealed that SIN may aid in the relief of the clinical symptoms of RA. Guo et al. explored the potential targets underlying the effect of SIN on RA by utilizing a network pharmacology approach; sixty-seven potential targets of SIN and 3797 related targets involved in RA were subjected to network analysis, and the 20 intersection targets indicated the principal pathways linked to RA [﻿39﻿]. In vitro and in vivo studies by Shen et al. have shown that thermosensitive liposomes loaded with sinomenine hydrochloride (SIN-TSL) combined with microwave thermotherapy have superior anti-RA effects [﻿40﻿]. In our research, almost 60% of the systematic reviews were found to have good methodological quality, and these reviews showed that ZQFTN or SIN could improve clinical symptoms and delay disease progression in patients with RA. These findings suggest that clinical trials on SIN for the treatment of RA may be prove its effectiveness.

The following are strengths of our overview. On the one hand, we used well-validated and accepted guidelines to assess both reporting and methodological quality. With the completion of a comprehensive and detailed plan, a rigorous and clear search strategy, and a highly adopted assessment guideline, we identified systematic reviews on the use of ZQFTN or SIN for the treatment of RA efficiently and reliably. On the other hand, we used the AMSTAR 2 system for reporting systematic reviews; AMSTAR 2 is an updated version of the classical AMSTAR instrument, and it conforms well to the PICO framework on research issues, controls the details of included studies more strictly, and considers RoB in more detail [﻿27﻿]. Furthermore, the GRADE system is a validated scientific approach used to evaluate the quality of evidence.

Although we followed strict procedures in this overview, it still has some limitations. First, although a predefined search strategy was used, we cannot guarantee that all relevant articles were included due to language limitations, which might have an effect on publication bias. Second, the methodological tools and reporting guidelines adopted in our study might not cover all details specific to systematic reviews and meta-analyses regarding RA. Third, the overall quality was not evaluated because we believed it would be sufficient to reflect the quality of each item instead of the overall quality. In addition, we used AMSTAR 2, released in 2017, whereas the included studies were published between 2008 and 2016, and no new study has been reported in the past 3 years, which may lead to bias. Last but not least, there are many other approaches that can be used to identify quality metrics, such as the journal impact factor, h-index, and other indicator systems [﻿41﻿, 42]. The impact factors of the eight studies were not satisfactory, which may also lead to certain publication bias and partiality.

## Conclusion

We collected 8 systematic reviews and meta-analyses published from database inception to July 2019 and assessed their methodological and reporting quality and quality of evidence. The average methodological quality score was 6.625, and the average reporting score was 17.69. In addition, 58% (*n* = 35, 35/61) of the outcome indicators had limitations based on the GRADE table. The reporting and methodological quality of the included meta-analyses and systematic reviews were less than optimal, which indicates that researchers should undergo additional training and follow the AMSTAR 2 scale, PRISMA statement and GRADE to design high-quality studies in the future. This procedure will provide better suggestions for the clinical treatment of RA.

## Supplementary information

**Additional file 1: Supplementary Table 1.** Search strategy for PubMed and the Cochrane Library. **Supplementary Table 2.** Search strategy for CNKI, Wanfang and VIP.

## Data Availability

The data sets supporting the conclusions of this article are included within the article.

## Abbreviations

SIN: Sinomenine
NSAIDs: Nonsteroidal anti-inflammatory drugs
NIP: Number of improved patients
NRP: Number of rheumatoid factor-disappeared patients
MS: Morning stiffness
PJ: Painful joint
SJ: Swollen joint
GS: Grip strength
ESR: Erythrocyte sedimentation rate
CRP: C-reactive protein
JTS: Joint tenderness score
AI: Articular index
ZQFTN: Zheng Qing Feng Tong Ning
MTX: Methotrexate
LEF: Leflunomide
D-pen: D-penicillamine
SSZ: Sulfasalazine
PED: Prednisone
ACR: American College of Rheumatology
RF: Rheumatoid factor
DAS28: Disease activity score-28
MeSH: Medical subject headings

## References

[1] Standish KA, Huang CC, Curran ME (2017). Comprehensive analysis of treatment response phenotypes in rheumatoid arthritis for pharmacogenetic studies. Arthritis Res Ther.

[2] Bader L, Gullaksen SE, Blaser N, Bader L, Brun M, Bringeland GH, Sulen A, Gjesdal CG, Vedeler C, Gavasso S (2019). Candidate Markers for Stratification and Classification in Rheumatoid Arthritis. Front Immunol.

[3] Almoallim H, Janoudi N, Alokaily F, Alzahrani Z, Algohary S, Alosaimi H, Attar S (2019). Achieving comprehensive remission or low disease activity in rheumatoid patients and its impact on workability - Saudi Rheumatoid Arthritis Registry. Open Access Rheumatol.

[4] Zhu LF, Feng YR, Xu DY (2018). Advances in research on quality of life in patients with rheumatoid arthritis. Rheum Arthritis.

[5] McInnes IB, Kim HY, Lee SH, Mandel D, Song YW, Connell CA, Luo Z, Brosnan MJ, Zuckerman A, Zwillich SH, Bradley JD (2014). Open-label tofacitinib and double-blind atorvastatin in rheumatoid arthritis patients: a randomised study. Ann Rheum Dis.

[6] Venkatesha SH, Rajaiah R, Berman BM, Moudgil KD (2011). Immunomodulation of Autoimmune Arthritis by Herbal CAM. Evid Based Complement Alternat Med.

[7] Boytsov NN, Bhattacharya R, Saverno K, Dixon L, Abbott PL, Zhang X, Gaich CL, Nair R (2019). Health Care Effect of Disease-Modifying Antirheumatic Drug Use on Patients with Rheumatoid Arthritis. J Manag Care Spec Pharm.

[8] Verhoeven MM, de Hair MJ, Tekstra J, Bijlsma JW, van Laar JM, Pethoe-Schramm A, Borm ME, Ter Borg EJ, Linn-Rasker SP, Teitsma XM, Lafeber FP, Jacobs JW, Welsing PM (2019). Initiating tocilizumab, with or without methotrexate, compared with starting methotrexate with prednisone within step-up treatment strategies in early rheumatoid arthritis: an indirect comparison of effectiveness and safety of the U-Act-Early and CAMERA-II treat-to-target trials. Ann Rheum Dis.

[9] Abbasi M, Mousavi MJ, Jamalzehi S, Alimohammadi R, Bezvan MH, Mohammadi H, Aslani S (2019). Strategies toward rheumatoid arthritis therapy; the old and the new. J Cell Physiol.

[10] Feng Z, Xu J, He G, Cao M, Duan L, Chen L, Wu Z (2016). The Efficacy and Safety of the Combination of Total Glucosides of Peony and Leflunomide for the Treatment of Rheumatoid Arthritis: A Systemic Review and Meta-Analysis. Evid Based Complement Alternat Med.

[11] Feng ZT, Xu J, He GC, Cai SJ, Li J, Mei ZG (2018). A systemic review and meta-analysis of the clinical efficacy and safety of total glucosides of peony combined with methotrexate in rheumatoid arthritis. Clin Rheumatol.

[12] Zhao XX, Peng C, Zhang H, Qin LP (2012). Sinomenium acutum: a review of chemistry, pharmacology, pharmacokinetics, and clinical use. Pharm Biol.

[13] Shen P, Tu S, Wang H, Qin K, Chen Z (2019). Simiao Pill Attenuates Collagen-Induced Arthritis in Rats through Suppressing the ATX-LPA and MAPK Signalling Pathways. Evid Based Complement Alternat Med.

[14] Zhou YY, Xia X, Peng WK, Wang QH, Peng JH, Li YL, Wu JX, Zhang JY, Zhao Y, Chen XM, Huang RY, Jakobsson PJ, Wen ZH, Huang QC (2018). The Effectiveness and Safety of Tripterygium wilfordii Hook. F Extracts in Rheumatoid Arthritis: A Systematic Review and Meta-Analysis. Front Pharmacol.

[15] Shen W, Guan YY, Wu RM, Liu LX, Li HD, Bao WL, Zhang YQ, Nandakumar KS, Shen XY (2019). Protective effects of Wang-Bi tablet on bone destruction in collagen-induced arthritis by regulating osteoclast-osteoblast functions. J Ethnopharmacol.

[16] Ernst E, Chrubasik S (2000). Phyto-anti-inflammatories: A Systematic Review of Randomized, Placebo-Controlled, Double-Blind Trials. Rheum Dis Clin N Am.

[17] Lu S, Wang Q, Li G, Sun S, Guo Y, Kuang H (2015). The treatment of rheumatoid arthritis using Chinese medicinal plants: From pharmacology to potential molecular mechanisms. J Ethnopharmacol.

[18] Moudgil KD, Berman BM (2014). Traditional Chinese medicine: potential for clinical treatment of rheumatoid arthritis. Expert Rev Clin Immunol.

[19] Huang L, Dong Y, Wu J, Wang P, Zhou H, Li T, Liu L (2017). Sinomenine-induced histamine release-like anaphylactoid reactions are blocked by tranilast via inhibiting NF-κB signaling. Pharmacol Res.

[20] Xu M, Liu L, Qi C, Deng B, Cai X (2008). Sinomenine versus NSAIDs for the treatment of rheumatoid arthritis: a systematic review and meta-analysis. Planta Med.

[21] Liu WW, Qian X, Ji W, Lu Y, Wei G, Wang Y (2016). Effects and safety of Sinomenine in treatment of rheumatoid arthritis contrast to methotrexate: a systematic review and Meta-analysis. J Tradit Chin Med.

[22] Feng ZT, Yang T, Hou XQ, Wu HY, Feng JT, Ou BJ, Cai SJ, Li J, Mei ZG (2019). Sinomenine mitigates collagen-induced arthritis mice by inhibiting angiogenesis. Biomed Pharmacother.

[23] Tong B, Yuan X, Dou Y, Wu X, Wang Y, Xia Y, Dai Y (2016). Sinomenine induces the generation of intestinal Treg cells and attenuates arthritis via activation of aryl hydrocarbon receptor. Lab Investig.

[24] Liu W, Zhang Y, Zhu W, Ma C, Ruan J, Long H, Wang Y (2018). Sinomenine Inhibits the Progression of Rheumatoid Arthritis by Regulating the Secretion of Inflammatory Cytokines and Monocyte/Macrophage Subsets. Front Immunol.

[25] Wei HH, Zhao XX, Chen L, Li SS, Song YL, Han TF, Ju DH, Hao BH (2013). Research progress and prospects of sinomenine in the treatment of rheumatoid arthritis. Chin J Tradit Chin Med Pharm.

[26] Wang Q, Li XK (2011). Immunosuppressive and anti-inflammatory activities of sinomenine. Int Immunopharmacol.

[27] Shea BJ, Reeves BC, Wells G, Thuku M, Hamel C, Moran J, Moher D, Tugwell P, Welch V, Kristjansson E, Henry DA (2017). AMSTAR 2: a critical appraisal tool for systematic reviews that include randomised or non-randomised studies of healthcare interventions, or both. BMJ..

[28] Moher D, Liberati A, Tetzlaff J, Altman DG (2009). Preferred reporting items for systematic reviews and meta-analyses: the PRISMA statement. BMJ..

[29] Mustafa RA, Santesso N, Brozek J, Akl EA, Walter SD, Norman G, Kulasegaram M, Christensen R, Guyatt GH, Falck-Ytter Y, Chang S, Murad MH, Vist GE, Lasserson T, Gartlehner G, Shukla V, Sun X, Whittington C, Post PN, Lang E, Thaler K, Kunnamo I, Alenius H, Meerpohl JJ, Alba AC, Nevis IF, Gentles S, Ethier MC, Carrasco-Labra A, Khatib R, Nesrallah G, Kroft J, Selk A, Brignardello-Petersen R, Schünemann HJ (2013). The GRADE approach is reproducible in assessing the quality of evidence of quantitative evidence syntheses. J Clin Epidemiol.

[30] Qi BJ, Liu CJ (2010). Chinese herbal medicine for the treatment of rheumatoid arthritis with methotrexate as a control. J Mod Integr Med.

[31] Zhang J, Wei W, Ji HY, Wang CM (2012). Systematic evaluation of the efficacy and safety of Zhengqing Fengtongning combined with western medicine in the treatment of rheumatoid arthritis. Tianjin Med.

[32] Li RC, Li J (2012). Zheng Qingfengtongning combined with methotrexate for the systematic evaluation of rheumatoid arthritis. Chin J Exp Tradit Med Form.

[33] Wang BX, Li X, Gu J, Huang HY, Wang JG, Fan FY, Cai X (2015). Systematic evaluation of the effectiveness and safety of Zhengqing Fengtongning in the treatment of rheumatoid arthritis. J Tradit Chin Med Univ Hunan.

[34] Chen XM, Huang RY, Huang QC, Chu YL, Yan JY (2015). Systemic Review and Meta-Analysis of the Clinical Efficacy and Adverse Effects of Zhengqing Fengtongning Combined with Methotrexate in Rheumatoid Arthritis. Evid Based Complement Alternat Med.

[35] Li X, Wang BX, Li RY, Wang SZ, Gu J, Wei YX, Yu HH, Peng L, Song HP, Huang HY, Fan FY, Cai X (2016). Systematic evaluation of randomized controlled trials of Zhengqing Fengtongning combined with chemical drugs for rheumatoid arthritis. Chin J Exp Tradit Med Form.

[36] Lunny C, Brennan SE, McDonald S, McKenzie JE (2016). Evidence map of studies evaluating methods for conducting, interpreting and reporting overviews of systematic reviews of interventions: rationale and design. Syst Rev.

[37] Manchikanti L, Benyamin RM, Helm S, Hirsch JA (2009). Evidence-based medicine, systematic reviews, and guidelines in interventional pain management: part 3: systematic reviews and meta-analyses of randomized trials. Pain Physician.

[38] Reza YN, Lohfeld L, Marin A, Hanneman R, Dobbins M (2017). Informing the implementation of evidence-informed decision making interventions using a social network analysis perspective; a mixed-methods study. BMC Health Serv Res.

[39] Guo X, Ji J, Feng Z, Hou X, Luo Y, Mei Z (2020). A network pharmacology approach to explore the potential targets underlying the effect of sinomenine on rheumatoid arthritis. Int Immunopharmacol.

[40] Shen Q, Zhang X, Qi J, Shu G, Du Y, Ying X (2020). Sinomenine hydrochloride loaded thermosensitive liposomes combined with microwave hyperthermia for the treatment of rheumatoid arthritis. Int J Pharm.

[41] Waltman L, Traag VA (2017). Use of the journal impact factor for assessing individual articles need not be wrong. arXiv preprint arXiv.

[42] Lucio BB, Lando T (2017). A theoretical model of the relationship between the h-index and other simple citation indicators. Scientometric..

