# Supplementary material for: Methodological and reporting quality evaluation of meta-analyses on the Chinese herbal preparation Zheng Qing Feng Tong Ning for the treatment of rheumatoid arthritis
Source: BMC Complement Med Ther. 2020 Jun 26;20:195. doi: 10.1186/s12906-020-02978-5 (PMC7318442; doi:10.1186/s12906-020-02978-5)
Supplement: Supplementary file 1 — Additional file 1: Supplementary Table 1. Search strategy for PubMed and the Cochrane Library. Supplementary Table 2. Search strategy for CNKI, Wanfang and VIP. [file 12906_2020_2978_MOESM1_ESM.docx]

**Supplementary Table 1 Search strategy for PubMed and the Cochrane Library**

| No. MeSH items |
| --- |
| #1 sinomenine ［Title/Abstract］ |
| #2 sinomenine preparation ［Title/Abstract］  #3 Zhengqing Fengtongning  #4 #1 OR #2 OR #3  #5 RA ［Title/Abstract］  #6 rheumatoid arthritis ［Title/Abstract］  #7 #5 OR #6  #8 meta-analysis ［Title/Abstract］  #9 systematic review ［Title/Abstract］  #10 #8 OR #9  #10 #4 AND #7 AND #10 |

**Supplementary Table 2 Search strategy for CNKI, Wanfang and VIP**

| No. Keywords (In Chinese) |
| --- |
| #1 Qing teng jian ［Title/Abstract］ |
| #2 Qing teng jian zhi ji ［Title/Abstract］  #3 Zheng qing feng tong ning  #4 #1 OR #2 OR #3  #5 Lei feng shi guan jie yan ［Title/Abstract］  #6 Lei feng shi xing guan jie yan［Title/Abstract］  #7 #5 OR #6  #8 meta fen xi［Title/Abstract］  #9 Xi tong ping jia ［Title/Abstract］  #10 Hui cui fen xi［Title/Abstract］  #11 #8 OR #9 OR #10  #12 #4 AND #7 AND #11 |
